# A turning point for youth HIV care in the United States: tailored strategies for adolescents and young adults

**DOI:** 10.1080/09581596.2026.2697406

**Published:** 2026-07-16

**Authors:** Omar Martinez, Maria Trent, Sybil Hosek, Jose Bauermeister, Julio Nazario-Valle, Rahim Miller, Larissa Jennings Mayo-Wilson, Zoë Njemanze, Jim Pickett, Michelle Ogle, Abigail English, Adrian Williams, Judah Dean, Patricia Emmanuel, Tina Simpson, Kristina Claude, Lisa Hightow-Weidman

**Affiliations:** a College of Medicine, University of Central Florida, Orlando, FL, USA;; b John Hopkins Bloomberg School of Public Health, John Hopkins University, Baltimore, MD, USA;; c College of Medicine, University of Illinois Chicago, Chicago, IL, USA;; d School of Nursing, University of Pennsylvania, Philadelphia, PA, USA;; e Volusia County Schools, DeLand, FL, USA;; f School of Nursing, Florida State University, Tallahassee, FL, USA;; g Gillings School of Global Public Health, University of North Carolina, Chapel Hill, NC, USA;; h Jim Pickett, Chicago, IL, USA;; i Department of Pediatrics, Albert Einstein College of Medicine, New York, NY, USA;; j Abigail English, Chapel Hill, NC, USA;; k College of Medicine, University of South Florida, Tampa, FL, USA;; l School of Medicine, Tulane University, New Orleans, LA, USA

**Keywords:** Adolescents and young adults, youth HIV prevention, HIV continuum of care, implementation science, ending the HIV epidemic

## Abstract

Adolescents and young adults (AYAs; ages 13–24) remain an underserved and disproportionately affected population within the United States (U.S.). HIV epidemic. Despite overall national declines in HIV incidence, youth continue to account for approximately one in five new infections, with persistent disparities among Black and Latino youth, other structurally vulnerable youth, and those living in the Southern U.S. These inequities reflect structural barriers and developmental mismatches between youth needs and adult-oriented HIV prevention and care systems. This commentary examines the evolving landscape of HIV among AYAs, highlighting persistent gaps in testing, linkage, retention, and viral suppression. Drawing on national surveillance data and a case example from Orange County, Florida, we illustrate how developmental vulnerability, stigma, and fragmented systems undermine engagement across the HIV care continuum. We highlight emerging opportunities, including long-acting injectable antiretroviral therapy and PrEP, digital health tools, and youth-focused case management, and emphasize the role of implementation science frameworks in adapting and sustaining these innovations. We argue that meaningful progress will require embedding youth voices in program design, addressing structural determinants of health through legal aid, and committing to sustained investment in youth-centered strategies. Reimagining the HIV response with adolescents and young adults at the center is essential to achieving progress toward national and global HIV targets.

## A turning point for youth HIV care

In the United States (U.S.), adolescents and young adults (AYAs), defined as individuals aged 13–24 years, remain disproportionately affected by HIV and continue to experience substantial gaps across the HIV prevention and care continuum despite national progress. In 2024, 765 youth (11.2%) received an early (Stage 0) HIV diagnosis. Among AYAs newly diagnosed with HIV, 6,823 (82.0%) were linked to HIV medical care within one month of diagnosis, a rate lower than the 83.1% observed among all 38,434 individuals aged 13 years and older diagnosed with HIV in the U.S. in 2024, suggesting persistent challenges in timely engagement in care among younger populations. Furthermore, only 73.0% of AYAs achieved viral suppression within six months of diagnosis, highlighting ongoing gaps in retention and treatment success across the HIV care continuum. Among the 28,433 AYAs living with diagnosed HIV, 82.2% received HIV medical care and 70.7% achieved viral suppression (Centers for Disease Control and Prevention, 2026). These disparities are even more alarming when compared with the broader population of people living with HIV. Newly released national surveillance data indicate that AYAs are more than three times as likely to be unaware of their HIV status (44% vs. 13%), substantially more likely to be disengaged from care (55% vs. 34%), and markedly less likely to achieve viral suppression (43% vs. 63%) (Centers for Disease Control and Prevention, 2026).

Marked racial and ethnic disparities further characterize HIV outcomes among young people. In 2024, viral suppression rates among youth aged 13–24 years ranged from 67.5% among Black/African American youth, the lowest of any racial or ethnic group, to 75.5% among Asian youth, the highest. Viral suppression rates were 71.9% among American Indian/Alaska Native youth, 74.2% among Hispanic/Latino youth, 75.0% among Native Hawaiian and Other Pacific Islander youth, 73.4% among White youth, and 74.5% among multiracial youth (Centers for Disease Control and Prevention, 2026). See Table 1.

Significant geographic disparities continue to characterize the HIV epidemic in the United States. The South bears a disproportionate share of the national HIV burden, accounting for more than half of all new HIV diagnoses despite comprising a smaller proportion of the U.S. population. CDC data demonstrate that the South also lags behind other regions across key HIV care continuum outcomes. Among the 38,434 individuals aged 13 years and older diagnosed with HIV in 2024, 83.1% were linked to HIV medical care within one month of diagnosis nationally; however, linkage to care was lowest among individuals residing in the South (81.5%). Similarly, while 71.1% of individuals diagnosed with HIV achieved viral suppression within six months of diagnosis nationwide, the South again had the lowest rate of viral suppression (69.2%) (Centers for Disease Control and Prevention, 2026). These findings highlight persistent regional inequities in HIV outcomes and suggest that adolescents and young adults living in Southern states face overlapping social, economic, legal, and healthcare barriers that may increase vulnerability to HIV acquisition and hinder timely engagement in care and sustained viral suppression.

The urgency of addressing these disparities is further reinforced by mathematical modeling studies demonstrating that recent progress remains fragile; reductions in sustained investments, including those supporting the Ending the HIV Epidemic initiative, could reverse gains and lead to renewed increases in HIV incidence, particularly among populations and regions already experiencing a disproportionate HIV burden (Baugher et al., 2025; Fauci et al., 2019; McCree et al., 2020; Stover et al., 2016).

AYA face unique challenges that heighten their vulnerability to HIV acquisition and undermine outcomes across the HIV care continuum. Limited testing opportunities and delayed diagnoses leave many unaware of their status (Reid et al., 2017). Developmentally normative behaviors including exploration of identity, peer influences, and experimentation and risk-taking, coupled with persistent stigma, discrimination, and fragmented systems of care, particularly during transitions from pediatric to adult services, weaken engagement (Bauermeister et al., 2009; Hussen et al., 2019; Murray et al., 2018; Perger et al., 2025; Ritchwood et al., 2020). These factors create a fragile care environment in which youth are less likely than older adults to be diagnosed, linked to care, retained in treatment, or to achieve viral suppression.

The U.S. is at a critical turning point for youth HIV prevention and care, marked by converging policy momentum, biomedical innovation, and growing recognition of persistent structural inequities affecting AYAs. Recent advances, including highly effective biomedical prevention tools, expanded telehealth delivery, and national initiatives aimed at ending the HIV epidemic, have created unprecedented opportunities to reduce HIV incidence among youth. At the same time, surveillance data continue to demonstrate disproportionate HIV burden, suboptimal engagement across the care continuum, and widening health disparities among Black, Latino, and other structurally vulnerable youth, underscoring the fragility of recent epidemiologic gains. Without intentionally youth-centered strategies, defined as approaches that are developmentally responsive, culturally affirming, trauma-informed, and structurally adaptive, progress risks stagnation or reversal. Youth-centered HIV strategies differ fundamentally from traditional adult-oriented models by accounting for normative developmental processes (e.g., increasing autonomy, identity exploration, and transitions in care responsibility), addressing stigma and discrimination, and reducing structural barriers such as fragmented systems of care, insurance instability, and limited access to confidential services. Drawing on recent policy developments, biomedical advances, and emerging community-level data, this commentary underscores the urgency of sustained investment, evidence-based interventions, and systemic reform that place AYAs at the center of HIV prevention, care, and treatment, where they have long been underrepresented despite bearing a disproportionate share of risk.

## Case example: Orange County, Florida – persistent gaps in HIV prevention and care for adolescents and young adults (ages 13–24)

Orange County, Florida, remains a critical epicenter of the HIV epidemic in Central Florida and illustrates persistent challenges affecting AYAs. According to the 2025 Integrated Epidemiological Profile, 256 AYAs aged 13–24 years were living with HIV in 2024, including 34 adolescents aged 13–19 years and 222 young adults aged 20–24 years (Florida Department of Health in Orange County, 2025). Orange County, Florida, remains a critical epicenter of the HIV epidemic in Central Florida and illustrates persistent challenges affecting adolescents and young adults (AYAs). According to the 2025 Integrated Epidemiological Profile, 256 AYAs aged 13–24 years were living with HIV in 2024, including 34 adolescents aged 13–19 years and 222 young adults aged 20–24 years. Although AYAs represent a relatively small proportion of all persons living with HIV in Orange County, this developmental period represents a critical window for prevention, early diagnosis, linkage to care, and sustained engagement in treatment. Important differences emerged across the HIV care continuum. Among adolescents aged 13–19 years living with HIV, 85.3% were documented as in care, while 14.7% were not in care. Retention in care, defined by ongoing engagement in HIV medical care during the surveillance period, was lower (70.6%), suggesting that some youth who initially access care do not remain consistently engaged. Overall viral suppression among youth ages 13–19 was 82.4%, and viral suppression reached 96.6% among those actively in care, indicating that treatment outcomes are excellent when adolescents remain connected to services. However, 17.6% had no documented viral load during the surveillance period, suggesting potential gaps in monitoring or care engagement. Similar challenges were observed among young adults aged 20–24 years. Although 84.7% were documented as in care, only 76.1% were retained in care. Overall viral suppression was 69.8%, substantially lower than among adolescents, although 82.4% of young adults engaged in care achieved viral suppression. As with adolescents, 17.6% had no documented viral load, indicating potential interruptions in monitoring or treatment engagement (Florida Department of Health in Orange County, 2025). See Table 2.

Taken together, the Orange County data highlight a clear and urgent need for youth-centered, developmentally responsive, and structurally adaptive HIV prevention and care strategies. Despite being a priority Ending the HIV Epidemic jurisdiction, limited research has examined HIV among AYAs in Orlando and Orange County (Balán et al., 2024). The limited Florida-based literature has primarily focused on AYAs in Miami and South Florida (Chutuape et al., 2010; LaLota et al., 2005; Malow et al., 2009; McCollister et al., 2014; Prado et al., 2010). Although biomedical advances have transformed HIV prevention and treatment, AYAs continue to experience lower retention in care and gaps in viral load monitoring. Without sustained investments in youth-friendly prevention, testing, and care models, alongside efforts to reduce stigma, strengthen transitions from pediatric to adult care, and address social and structural barriers, recent epidemiologic gains may stall or reverse. Importantly, the patterns observed in Orange County are not unique; rather, they reflect persistent and well-documented disparities across the HIV care continuum among adolescents and young adults throughout the United States (Lall et al., 2015; Lightfoot, 2012; Morris et al., 2006; Perger et al., 2025).

## Innovations and emerging opportunities

Addressing persistent gaps in HIV prevention and care for AYA requires more than incremental improvement, it demands a strategic shift toward tailored, youth-centered innovations that are developmentally responsive and structurally adaptive. Emerging biomedical advances, digital health tools, and data-informed care models offer unprecedented opportunities to reconfigure how services are delivered and sustained for youth. However, realizing their full potential depends on intentionally integrating these innovations into systems of care in ways that align with young people’s lived realities. Effective strategies must embed transition preparedness early in adolescent HIV prevention and care, pairing clinical services with insurance navigation, peer-based support, digital engagement and adherence tools, legal aid, and flexible delivery models such as telehealth and long-acting therapies. Critically, translating innovation into impact requires the deliberate application of implementation science, co-designing interventions with youth, strengthening provider capacity, embedding trauma-informed and culturally responsive practices, and systematically documenting adaptations to improve local fit while preserving effectiveness. Without this intentional and coordinated approach, promising innovations risk remaining siloed or inaccessible, and adolescents and young adults will continue to be marginalized within the HIV response.

## Biomedical advances: long-acting therapies for adolescents and young adults in the United States

The rapid expansion of long-acting HIV therapies for both treatment and prevention represents a critical opportunity to improve outcomes for AYAs in the U.S. Long-acting injectable antiretroviral therapy (LAI-ART) and long-acting pre-exposure prophylaxis (PrEP) reduce the burden of daily oral dosing, mitigate stigma associated with pill-taking, and offer discreet, low-burden options that align with the developmental needs of youth. These attributes are particularly salient for AYAs navigating confidentiality concerns, inconsistent routines, and transitions in healthcare responsibility.

Early clinical and real-world data demonstrate strong potential. Studies of LAI-ART conducted primarily among adults have shown that nearly 90% of participants remain virologically suppressed six months after initiation (Nachega et al., 2023), with observational data indicating that over 85% of individuals with prior adherence challenges maintain viral suppression at 48 weeks (Hastie et al., 2025). Emerging evidence among adolescents and young adults is similarly encouraging. Findings from the IMPAACT 2017/MOCHA study demonstrated high acceptability, favorable safety profiles, and sustained viral suppression among adolescents receiving long-acting injectable cabotegravir and rilpivirine, supporting the feasibility of LAI-ART as a youth-centered treatment strategy (Gaur et al., 2026; Lowenthal et al., 2024). These data suggest that long-acting treatment may be especially beneficial for adolescents and young adults facing structural, developmental, or behavioral barriers to daily adherence, provided that delivery models address appointment adherence, continuity of care, and transitions between pediatric and adult healthcare systems. Parallel advances in HIV prevention further underscore the importance of expanding choice for AYAs. Long-acting injectable PrEP among adults has demonstrated superior efficacy compared to daily oral PrEP (Landovitz et al., 2021), and emerging evidence suggests high levels of interest, acceptability, and preference for long-acting prevention modalities among youth (Abrams et al., 2022; Kapogiannis et al., 2018; Lorenzetti et al., 2023; Lunkuse et al., 2025; Magno et al., 2025; Okafor et al., 2024). These findings highlight the potential of long-acting PrEP to address barriers associated with daily medication adherence and expand prevention choices for AYAs. However, achieving sustained prevention coverage will require a multifaceted, youth-responsive prevention portfolio that recognizes the diverse needs and preferences of young people. Evidence from reproductive health demonstrates that increasing method choice improves uptake, satisfaction, and persistence, reinforcing the importance of avoiding one-size-fits-all approaches to HIV prevention (Bertrand et al., 2020; Ross and Stover, 2013).

Together, these advances highlight the promise of biomedical innovation to reduce HIV-related disparities among U.S. AYAs. Realizing this potential, however, will require intentional, youth-centered implementation strategies that prioritize choice, accessibility, confidentiality, and continuity across care settings.

## Digital frontiers and predictive analytics

Digital health interventions, including telemedicine, mobile health (mHealth) applications, text messaging, social media platforms, and AI-enabled navigation tools, offer promising strategies to address gaps in HIV prevention and care among AYAs (Barman-Adhikari et al., 2016; Dowshen et al., 2015; Gurung et al., 2023; Ogunlana et al., 2025). AYAs may particularly benefit from interventions that are developmentally appropriate, culturally responsive, and integrated into their daily lives. Systematic reviews suggest that digital interventions can improve HIV-related knowledge, testing uptake, ART adherence, and access retention in care in general health for AYA, particularly when they incorporate peer support, personalized feedback, and interactive features (Mulawa et al., 2018; Whitehead et al., 2024). However, sustained engagement remains a challenge, underscoring the importance of youth-centered design and implementation strategies (Brasileiro et al., 2025).

Telehealth has expanded access to HIV services by reducing barriers related to transportation, stigma, and provider shortages, with evidence supporting improvements in linkage to care, retention, and ART adherence (Krebs et al., 2025). Among AYAs, studies have also reported high acceptability and satisfaction with telehealth, as well as strong interest in continued use due to its convenience, privacy, and potential to increase access to HIV care and support services (Barr et al., 2025; Koay et al., 2024; Wootton et al., 2019). Similarly, mHealth interventions, including smartphone applications, SMS reminders, and digital adherence tools, have demonstrated promise for supporting self-management and care engagement among youth, particularly when grounded in behavioral theory and co-designed with intended users (Mulawa et al., 2018; Schnall et al., 2026). Advances in artificial intelligence (AI) and predictive analytics are also transforming HIV prevention and care delivery (Beegle et al., 2025; Pant Pai et al., 2025; Zhang et al., 2026). For HIV prevention, electronic health record (EHR)-based machine learning models can identify individuals at elevated risk for HIV acquisition who may benefit from proactive PrEP outreach, improving the precision of prevention efforts (Liu et al., 2025; Marcus et al., 2019). For HIV care, predictive models integrating clinical, behavioral, and social data can identify patients at risk for missed visits, treatment interruptions, or loss to follow-up, enabling targeted navigation and retention interventions (Oliwa et al., 2021; Ridgway et al., 2025), with emerging evidence supporting their utility among AYAs (Najjuuko et al., 2025).

The growing use of digital and AI-enabled tools also raises important concerns related to privacy, confidentiality, and equity. Adolescents face unique risks of inadvertent disclosure through shared devices, family accounts, and limited private spaces for telehealth visits (Whitehead et al., 2024). In addition, predictive algorithms may inadvertently perpetuate existing inequities if they incorporate biased data or social determinants that reflect structural disadvantage. Experts therefore recommend transparent data governance, robust privacy protections, and routine auditing of AI systems for fairness (Mayur, 2026; Rajgopal and Yadav, 2025).

Despite their promise, digital technologies alone are unlikely to eliminate disparities in HIV outcomes. Their impact will depend on effective integration within healthcare, educational, and social service systems, as well as implementation strategies that promote access, sustained engagement, and long-term scalability among AYAs.

## Where youth live and work

Beyond healthcare settings, schools, colleges, and community-based organizations represent critical developmental contexts in which AYAs navigate identity formation, relationships, and health-related decision-making. These settings shape access to sexual health information, social support, healthcare services, and opportunities for HIV prevention and care, yet they remain underutilized as sites for HIV intervention. Because AYAs spend much of their time within educational and community settings, these environments provide important opportunities to reach youth who may have limited engagement with traditional healthcare systems and to address barriers before they result in care disengagement or increased HIV vulnerability (Hosek and Pettifor, 2019; Serrano et al., 2025).

Evidence suggests that school-based health centers can improve access to preventive health services, sexual and reproductive healthcare, sexually transmitted infection (STI) screening, and health education among adolescents, particularly those from underserved communities. School-based programs that integrate HIV education, testing, and referral services have also demonstrated promise for increasing HIV testing uptake and reducing barriers to care among youth who may not otherwise engage with healthcare systems (Arenson et al., 2019; Borkowski et al., 2023; Ethier et al., 2011).

Community-based organizations play an equally important role in extending the reach of HIV prevention and care beyond clinical settings. Community-led interventions, peer navigation programs, youth-serving organizations, and social network approaches have been shown to increase HIV testing, linkage to care, prevention service utilization, and health literacy among adolescents and young adults, particularly among populations disproportionately affected by HIV (Hosek and Pettifor, 2019). Community-based programs are often uniquely positioned to address stigma, build trust, and provide culturally responsive services for sexual and gender minority youth, youth of color, and youth experiencing housing instability or other structural vulnerabilities. Emerging evidence further suggests that community-engaged and peer-led interventions can successfully leverage social networks to promote HIV prevention behaviors among highly vulnerable youth populations (Lee et al., 2025; Lightfoot et al., 2022). For example, studies among youth experiencing homelessness have demonstrated that peer-based interventions implemented through community settings can improve HIV testing and prevention outcomes (Lee et al., 2025; MacEntee et al., 2022; Podschun, 1993; Rice et al., 2012).

Taken together, the evidence suggests that effective HIV prevention and care for AYAs requires moving beyond clinic-centered approaches toward multi-sector strategies that engage educational institutions and community organizations as active partners in promoting health. Integrating HIV services within these settings may extend the reach of clinical innovations, strengthen community support systems, and address structural barriers that contribute to disparities in HIV outcomes among young people.

### Implementation science frameworks for advancing HIV care among adolescents and young adults

Implementation science frameworks such as Reach, Effectiveness, Adoption, Implementation, and Maintenance (RE-AIM) (Brant et al., 2020; Glasgow et al., 1999; Paone et al., 2025; Salinas and Valenzuela, 2024) and the Consolidated Framework for Implementation Research (CFIR) (Baron et al., 2024; Gomez et al., 2021; Paone et al., 2025; Sabri et al., 2026) provide structured guidance for adapting, evaluating, and scaling HIV innovations across varied service settings. Complementing these approaches, Proctor implementation outcomes framework (Proctor et al., 2011), including feasibility, acceptability, appropriateness, adoption, fidelity, penetration, and sustainability, offers essential metrics for evaluating HIV programs beyond traditional clinical endpoints. More recently, the Health Equity Implementation Framework (HEIF) (Woodward et al., 2019) has advanced the field by explicitly integrating determinants of health disparities and community priorities, into implementation planning and evaluation. This equity-centered lens is particularly salient for AYAs, who navigate intersecting developmental, structural, and social barriers, including stigma, discrimination, confidentiality concerns, and transitions in care.

Despite their relevance, these frameworks have been infrequently applied in HIV research focused on AYAs. Youth-oriented HIV studies continue to prioritize efficacy and effectiveness outcomes, often with limited attention to the multilevel contextual and related factors that shape real-world implementation and sustainability. Tailoring implementation frameworks for AYAs requires centering youth perspectives, ensuring developmental appropriateness, and embedding culturally responsive and trauma-informed strategies that reflect the realities of disclosure, stigma, and fragmented systems of care. Without such intentional adaptation, implementation efforts risk perpetuating the very health disparities they seek to address.

### Medical-legal partnerships as structural interventions

Medical-legal partnerships (MLPs) are increasingly recognized as structural health interventions that address upstream legal and social determinants of health that shape disease risk, care engagement, and long-term outcomes (Fuller et al., 2020; Girard et al., 2023; Klein et al., 2013; Spiegel et al., 2023). By embedding legal services within healthcare and community-based settings, MLPs intervene on health-harming legal needs related to housing, income, education, and access to public benefits, domains that traditional clinical care alone cannot address (Hemeida and Wong, 2022).

A growing body of research demonstrates the effectiveness of MLPs in addressing a wide range of health conditions, including HIV (Martinez et al., 2025, 2026). In particular, studies have documented the direct impact of legal aid on HIV care continuum outcomes when MLPs are embedded within HIV/AIDS service organizations and Federally Qualified Health Centers (FQHCs) (Martinez et al., 2022, 2026). These impacts include improvements in appointment adherence, retention in care, and viral load suppression among adults living with HIV (Jaén et al., 2024; Martinez et al., 2026). Legal interventions addressing housing instability, benefit denials, discrimination, and insurance access have been shown to stabilize patients’ lives in ways that enable sustained engagement in HIV care. Despite this evidence (Benfer et al., 2018; Ryan et al., 2012; Setrini, 2023; Spiegel et al., 2023), MLPs remain underexplored among AYA, including those at risk for or living with HIV. This gap is notable given that AYA disproportionately experience legal and structural barriers, such as housing insecurity, family instability, school disciplinary actions, and lack of access to public benefits, that directly undermine health, educational attainment, and care engagement. Future research should intentionally adapt and test MLP models for AYA, with attention to developmental appropriateness, confidentiality, consent, and cross-sector coordination.

Importantly, there are existing MLP models beyond traditional healthcare settings that offer valuable lessons for AYA-focused adaptation. For example, school-based health centers with integrated MLPs have demonstrated promise in supporting teen parents and low-income families by facilitating access to public benefits such as Temporary Assistance for Needy Families (TANF) and Supplemental Nutrition Assistance Program (SNAP), as well as ensuring emergency access to health insurance and other safety-net supports (The National Center for Medical-Legal Partnership, 2018). These models illustrate how MLPs can be embedded in trusted, youth-facing institutions and aligned with educational and social service systems. During community-engaged discussions between the first author and a Florida educator focused on identifying opportunities to adapt MLP approaches within youth-serving educational settings, the educator highlighted several areas where MLP principles could be integrated into existing school systems. As the educator noted, *‘I see tremendous potential for us to adapt elements of this model in ways that are sustainable and appropriate for our context. Specifically, I believe we can focus on areas like housing safety and stability, addressing issues such as unsafe living conditions, utility shut*-*offs, and environmental hazards that directly impact student health and attendance, while also connecting families to existing public benefits they are entitled to but may not know how to access. In addition, providing special education advocacy and Individualized Education Plan (IEP) support for families navigating complex systems, along with crisis intervention to prevent homelessness or family disruption that would disrupt schooling, represents a critical opportunity for impact’*. While illustrative rather than systematic data, this stakeholder perspective highlights how MLPs may be tailored to address the interconnected legal, educational, and social needs of youth and families, reinforcing their potential as developmentally responsive structural interventions.

Together, these insights underscore the potential of MLPs to function as developmentally responsive, youth-oriented structural interventions for AYA. By addressing legal needs that intersect with housing, education, family stability, and economic security, AYA-focused MLPs may not only improve health outcomes, including HIV prevention and care, but also strengthen educational continuity and long-term well-being.

### Youth voices and community engagement

The inclusion of youth voices in shaping HIV prevention and care strategies is essential. Programs co-designed and monitored by youth advisory boards foster trust, relevance, and sustainability (Chidester et al., 2023; Geffen et al., 2023; Hosek et al., 2008; Johnson et al., 2024). Peer-led outreach and education can further strengthen engagement, particularly when programs are linguistically and culturally tailored. However, a persistent challenge is sustainability after research funding ends. Too often, youth-centered interventions dissolve when grants conclude, leaving communities without the necessary resources or infrastructure to sustain themselves. Building models that emphasize community ownership, capacity building, and integration into existing health systems is essential for achieving a lasting impact.

To move beyond symbolic participation, youth engagement must be treated as a structured research practice rather than a single activity. Evidence from community-engaged frameworks demonstrates that equitable partnerships improve study relevance, recruitment, retention, and dissemination (Boyer et al., 2016; Nwaozuru et al., 2025). Mechanisms that strengthen engagement include early involvement of youth in protocol development, transparent communication about how input will be used, and feedback loops to demonstrate impact. Appropriate compensation and professional recognition are also essential components of ethical engagement.

Within the Adolescent Medicine Trials Network for HIV Interventions (ATN), the Subject Matter Research Consultant (SMRC) model illustrates how youth engagement can be institutionalized through formal infrastructure rather than reliance on individual goodwill or intention alone (Adolescent Medicine Trials Network for HIV Interventions, 2026; Institute on Digital Health and Innovation, 2025). SMRCs serve as compensated contributors across protocol teams and Scientific Leadership Groups, providing lived experience expertise to inform study materials, recruitment strategies, implementation decisions, and dissemination efforts. More broadly, the SMRC model demonstrates that sustainable youth engagement requires dedicated resources, compensation, clearly defined roles, leadership support, and long-term partnership strategies embedded within research networks and health systems. As HIV prevention and care initiatives increasingly emphasize community engagement and implementation science, the SMRC model offers a promising framework for moving beyond tokenistic participation toward authentic youth partnership and co-leadership in research and program development.

## Reimagining youth HIV care at the center: a call to action

**Figure 1.**
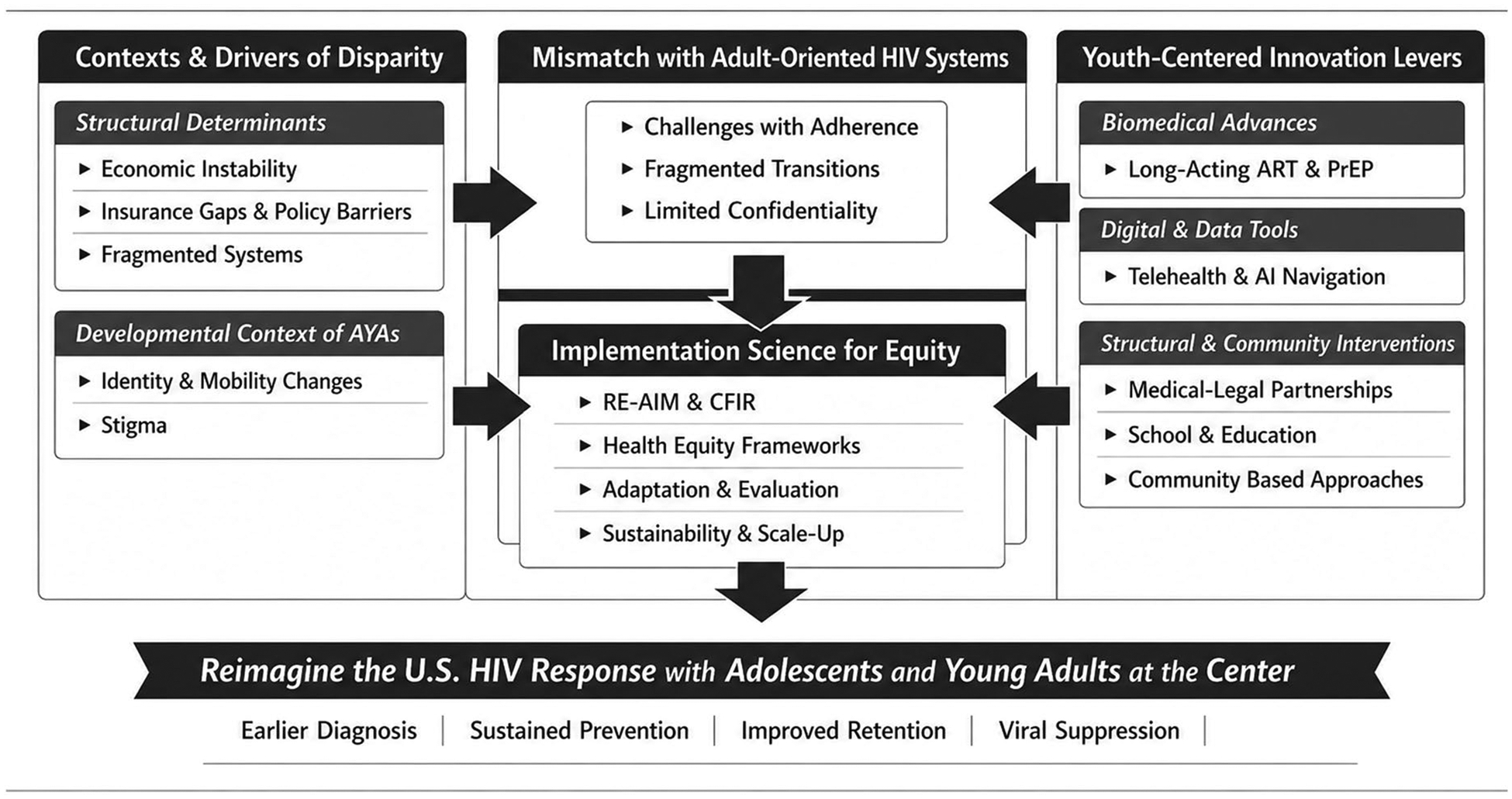
Conceptual framework illustrating how structural determinants of health, developmental vulnerabilities, and the mismatch between adolescent and young adult needs and adult-oriented HIV systems contribute to persistent gaps across the HIV prevention and care continuum. The framework highlights youth-centered biomedical, digital, and structural innovations, supported by implementation science and meaningful youth engagement, as critical levers for achieving equitable and sustained improvements in HIV prevention and care outcomes.

AYAs remain disproportionately burdened by HIV, yet their needs continue to be insufficiently prioritized in prevention, care, and policy responses. National and local epidemiologic data reveal persistent gaps across the HIV continuum, particularly among Black and Latino youth, other structurally vulnerable youth, and those living in the Southern United States, underscoring the urgency of action at this critical juncture. As illustrated in Figure 1, these disparities emerge from the interaction of structural determinants and developmental vulnerabilities within largely adult-oriented HIV systems that are often poorly aligned with the realities of youth. Although biomedical innovations, digital health tools, and youth-focused service models offer unprecedented opportunities to improve outcomes, innovation alone is insufficient without intentional strategies to ensure equitable implementation, scale, and sustainability.

Consistent with the framework depicted in Figure 1, we call on researchers, healthcare systems, policymakers, and funders to prioritize the following actions:

- Invest in youth-centered HIV prevention and care models that are developmentally responsive, culturally affirming, and tailored to the needs of diverse adolescent and young adult populations.
- Expand access to long-acting prevention and treatment options, including long-acting injectable PrEP and ART, while ensuring equitable implementation across settings serving structurally vulnerable youth.
- Strengthen transitions from pediatric to adult HIV care through coordinated, developmentally appropriate approaches that reduce care disruption and support long-term engagement.
- Integrate digital, community-based, and peer-led supports that complement clinical care and extend the reach of HIV prevention, treatment, and retention services.
- Address upstream structural determinants of health, including housing instability, transportation barriers, insurance gaps, stigma, legal needs, and economic insecurity that continue to shape HIV risk and care engagement.
- Institutionalize meaningful youth engagement by embedding youth as compensated partners in research, program design, implementation, evaluation, and policy development through clearly defined roles and long-term partnerships.
- Support implementation science and scale-up efforts to identify effective strategies, promote adaptation while maintaining fidelity, and generate evidence regarding what works, for whom, and under what conditions.

At this turning point, reimagining the HIV response with adolescents and young adults at the center is both a moral imperative and a public health necessity. Without decisive and coordinated action, recent gains risk stagnation or reversal. Consistent with rights-based approaches to HIV prevention and care (Barr et al., 2011), future efforts must ensure that young people have equitable access to high-quality, youth-friendly services while promoting autonomy, participation, confidentiality, and meaningful engagement in decisions that affect their health and well-being. With sustained investment and commitment, the U.S. has the opportunity to build a more equitable, effective, and youth-responsive HIV response, one capable of ending the epidemic for this generation and those that follow.

## Figures and Tables

**Table 1.** HIV care continuum and viral suppression disparities among AYAs in the United States, 2024.

Indicator	AYA (13–24 years)	Comparison
HIV care continuum		
Early (Stage 0) HIV diagnosis among newly diagnosed youth	11.2%	—
Linked to HIV care within 1 month of diagnosis	82.0%	83.1% (all persons ≥13 years)
Viral suppression within 6 months of diagnosis	73.0%	—
Received HIV medical care among persons living with diagnosed HIV	82.2%	—
Viral suppression among persons living with diagnosed HIV	70.7%	—
Unaware of HIV status	44.0%	13.0% (overall population)
Disengaged from care	55.0%	34.0% (overall population)
Virally suppressed	43.0%	63.0% (overall population)
Viral suppression by race/ethnicity	%	
Black/African American	67.5	Lowest
American Indian/Alaska Native	71.9	
White	73.4	
Hispanic/Latino	74.2	
Multiracial	74.5	
Native Hawaiian/Other Pacific Islander	75.0	
Asian	75.5	Highest

*Source*: CDC HIV Surveillance Report published 18th May 2026.

**Table 2.** HIV care continuum outcomes among AYAs living with HIV in Orange County, Florida, 2024.

HIV continuum indicator	Adolescents (13–19 years) (*n* = 34)	Young adults (20–24 years) (*n* = 222)
Living with HIV, *n*	34	222
In care, %	85.3%	84.7%
Out of care, %	14.7%	15.3%
Retained in care, %	70.6%	76.1%
Viral suppression, overall %	82.4%	69.8%
Viral suppression among those in care, %	96.6%	82.4%
No documented viral load, %	17.6%	17.6%
Newly diagnosed HIV cases, n	13	36

*Source*: Florida Department of Health, Bureau of Communicable Diseases, HIV/AIDS Section.

## Data Availability

This article is a commentary and does not report original empirical data. Materials supporting the perspectives dis-cussed in this commentary are available from the authors upon reasonable request.

## References

[R1] Abrams EJ, Capparelli E, Ruel T, & Mirochnick M. (2022). Potential of long-acting products to transform the treatment and prevention of human immunodeficiency virus (HIV) in infants, children, and adolescents. Clinical Infectious Diseases, 75(Suppl 4), S562–S570. 10.1093/cid/ciac754 36410381 PMC10200315

[R2] Adolescent Medicine Trials Network for HIV Interventions. (2026). ATN youth: Subject Matter Research Consultants (SMRC). https://atnresearch.org/atn-youth/?utm]

[R3] Arenson M, Hudson PJ, Lee N, & Lai B. (2019). The evidence on school-based health centers: A review. Global Pediatric Health, 6, 2333794X19828745. 10.1177/2333794X19828745 PMC6381423 30815514

[R4] Balán IC, Cheshure A, Green S, Coyle K, Cook C, Pooler-Burgess M, Wang Y, Morgan J, George A, & Naar S. (2024). Building an HIV learning health care community for youth in Florida: Opportunities and challenges. AIDS and Behavior, 28(3), 951–962. 10.1007/s10461-023-04201-1 37922033 PMC11068034

[R5] Barman-Adhikari A, Rice E, Bender K, Lengnick-Hall R, Yoshioka-Maxwell A, & Rhoades H. (2016). Social networking technology use and engagement in HIV-related risk and protective behaviors among homeless youth. Journal of Health Communication, 21(7), 809–817. 10.1080/10810730.2016.1177139 27337044 PMC5158181

[R6] Baron D, Leslie HH, Mabetha D, Becker N, Kahn K, & Lippman SA. (2024). Applying CFIR to assess multi-level barriers to PrEP delivery in rural South Africa: Processes, gaps and opportunities for service delivery of current and future PrEP modalities. Social Science & Medicine, 361, 117370. 10.1016/j.socscimed.2024.117370 39366151 PMC11554290

[R7] Barr D, Amon JJ, & Clayton M. (2011). Articulating a rights-based approach to HIV treatment and prevention interventions. Current HIV Research, 9(6), 396–404. 10.2174/157016211798038588 21999775 PMC3528010

[R8] Barr EA, (2025). Telehealth care for adolescents and young adults with HIV: Practices and perspectives from a national mixed-methods study of U.S. HIV care providers. MedRxiv: the preprint server for health sciences.

[R9] Bauermeister JA, Elkington K, Brackis-Cott E, Dolezal C, & Mellins CA. (2009). Sexual behavior and perceived peer norms: Comparing perinatally HIV-infected and HIV-affected youth. Journal of Youth and Adolescence, 38(8), 1110–1122. 10.1007/s10964-008-9315-6 19636775 PMC2769517

[R10] Baugher AR, Wejnert C, Kanny D, Broz D, Feelemyer J, Hershow RB, Burnett J, Chapin-Bardales J, Haynes M, Finlayson T, & Prejean J. (2025). Are we ending the HIV epidemic among persons who inject drugs?: Key findings from 19 US cities. AIDS, 39(12), 1813–1819. 10.1097/QAD.0000000000004249 40478920 PMC12424270

[R11] Beegle S, Gomez LA, Blackard JT, Yan B, Robertson J, Fedders KT, Horner S, Miller R, Rodriguez CR, Atreya A, & Brown JL. (2025). HIV prevention and treatment information from four artificial intelligence platforms: A thematic analysis. AIDS and Behavior, 29(11), 3394–3403. 10.1007/s10461-025-04786-9 40481266 PMC12500828

[R12] Benfer EA, Gluck AR, & Kraschel KL. (2018). Medical-legal partnership: Lessons from five diverse MLPs in New Haven, Connecticut. The Journal of Law, Medicine & Ethics, 46(3), 602–609. 10.1177/1073110518804210 30336104

[R13] Bertrand JT, Ross J, Sullivan TM, Hardee K, & Shelton JD. (2020). Contraceptive method mix: Updates and implications. Global Health, Science and Practice, 8(4), 666–679. 10.9745/GHSP-D-20-00229 33361234 PMC7784075

[R14] Borkowski V, Goddard A, & Gaffney B. (2023). School-based health centers: A concept analysis. Journal of Pediatric Health Care, 37(3), 269–278. 10.1016/j.pedhc.2022.11.005 36470799

[R15] Boyer CB, Walker BC, Chutuape KS, Roy J, & Fortenberry JD. (2016). Creating systems change to support goals for HIV continuum of care: The role of community coalitions to reduce structural barriers for adolescents and young adults. Journal of HIV/AIDS & Social Services, 15(2), 158–179. 10.1080/15381501.2015.1074977 PMC4880364 27239165

[R16] Brant AR, Dhillon P, Hull S, Coleman M, Ye PP, Lotke PS, Folan J, & Scott RK. (2020). Integrating HIV pre-exposure prophylaxis into family planning care: A RE-AIM framework evaluation. AIDS Patient Care and STDs, 34(6), 259–266. 10.1089/apc.2020.0004 32484743 PMC7262643

[R17] Brasileiro J, Queiroz A, Hightow-Weidman LB, & Muessig KE. (2025). Implementation strategies for digital HIV prevention and care interventions for youth: A scoping review. Current HIV/AIDS Reports, 22(1), 23. 10.1007/s11904-025-00732-5 40080278 PMC12004249

[R18] Centers for Disease Control and Prevention. (2026). National HIV prevention and care objectives: 2026 Update. Centers for Disease Control and Prevention.

[R19] Chidester AB, Johnson CJ, Lin H, Viera Corral R, Kools S, Ingersoll KS, Dillingham RA, Nijhawan AE, Taranova AG, & Taylor BS. (2023). Nothing about us without us: Involving youth living with HIV in a virtual advisory board. The Journal of Adolescent Health, 73(6), 1158–1161. 10.1016/j.jadohealth.2023.06.028 37665305 PMC11140764

[R20] Chutuape KS, Willard N, Sanchez K, Straub DM, Ochoa TN, Howell K, Rivera C, Ramos I, & Ellen JM. (2010). Mobilizing communities around HIV prevention for youth: How three coalitions applied key strategies to bring about structural changes. AIDS Education and Prevention, 22(1), 15–27. 10.1521/aeap.2010.22.1.15 20166784 PMC2850206

[R21] Dowshen N, Lee S, Matty Lehman B, Castillo M, & Mollen C. (2015). IknowUshould2: Feasibility of a youth-driven social media campaign to promote STI and HIV testing among adolescents in Philadelphia. AIDS and Behavior, 19 Suppl 2(0 2), 106–111. 10.1007/s10461-014-0991-9 25563502 PMC4495004

[R22] Ethier KA, Dittus PJ, DeRosa CJ, Chung EQ, Martinez E, & Kerndt PR. (2011). School-based health center access, reproductive health care, and contraceptive use among sexually experienced high school students. The Journal of Adolescent Health, 48(6), 562–565. 10.1016/j.jadohealth.2011.01.018 21575814

[R23] Fauci AS, Redfield RR, Sigounas G, Weahkee MD, & Giroir BP. (2019). Ending the HIV epidemic: A plan for the United States. JAMA, 321(9), 844–845. 10.1001/jama.2019.1343 30730529

[R24] Florida Department of Health in Orange County. (2025). HIV statistics, Orange County: Surveillance data 2020–2024 [Unpublished raw data]. Florida Department of Health in Orange County.

[R25] Fuller SM, Steward WT, Martinez O, & Arnold EA. (2020). Medical-legal partnerships to support continuity of care for immigrants impacted by HIV: Lessons learned from California. Journal of Immigrant and Minority Health, 22(1), 212–215. 10.1007/s10903-019-00919-0 31332651 PMC10729648

[R26] Gaur AH, Baltrusaitis K, Capparelli EV, Moye JH, Yin DE, Masheto G, Buisson S, Harrington CM, Marzinke MA, Lowenthal ED, Scheckter R, Ace A, Ward S, Milligan R, Whitson K, Huang J, Cheung SYA, Best BM, Townley E, … Stotz C. (2026). Safety, antiviral activity, and pharmacokinetics of long-acting injectable cabotegravir-rilpivirine in virologically suppressed adolescents living with HIV-1 (IMPAACT 2017/MOCHA): 48-Week results of a multinational, phase 1/2, single-arm study. The Lancet HIV, 13(2), e85–e94. 10.1016/S2352-3018(25)00242-5 41547359 PMC13292839

[R27] Geffen SR, Wang T, Cahill S, Fontenot HB, Conron K, Wilson JM, Avripas SA, Michaels S, Johns MM, & Dunville R. (2023). Recruiting, facilitating, and retaining a youth community advisory board to inform an HIV prevention research project with sexual and gender minority youth. LGBT Health, 10(2), 93–98. 10.1089/lgbt.2022.0213 36637887

[R28] Girard VW, Cannon YZ, Perry DF, & Moore ES. (2023). Leveraging academic-medical legal partnerships to advance health justice. The Journal of Law, Medicine & Ethics, 51(4), 798–809. 10.1017/jme.2024.5 PMC10937176 38477286

[R29] Glasgow RE, Vogt TM, & Boles SM. (1999). Evaluating the public health impact of health promotion interventions: The RE-AIM framework. American Journal of Public Health, 89(9), 1322–1327. 10.2105/ajph.89.9.1322 10474547 PMC1508772

[R30] Gomez SA, Martinez-Cajas JL, Mueses HF, Alvarado-Llano B, Galindo X, Camargo P, Martinez E, Torres JA, & Arrivillaga M. (2021). The CFIR application on PrEP implementation: Facilitators and recommendations by HIV-care clinic administrators in Colombia. Journal of the International AIDS Society, 24(Supplement 1), 192.

[R31] Gurung S, Jones SS, Mehta K, Budhwani H, MacDonell K, Belzer M, & Naar S. (2023). Examining recruitment strategies in the enrollment cascade of youth living with HIV: Descriptive findings from a nationwide web-based adherence protocol. JMIR Formative Research, 7(1), e40077. 10.2196/40077 36745773 PMC10131637

[R32] Hastie E, Hill L, Bamford L, Karim A, & Martin TCS. (2025). Long-acting injectable HIV therapy outcomes among persons with HIV who have adherence challenges to oral antiretroviral therapy. Clinical Infectious Diseases, 81(3), 543–546. 10.1093/cid/ciaf120 40096545 PMC12497944

[R33] Hemeida S, & Wong S. (2022). Integrating legal services to improve behavioral health, a challenge met from a different angle. Families, Systems & Health: The Journal of Collaborative Family Healthcare, 40(4), 609–612. 10.1037/fsh0000773 36508636

[R34] Hosek SG, Harper GW, Lemos D, & Martinez J. (2008). An ecological model of stressors experienced by youth newly diagnosed with HIV. Journal of HIV/AIDS Prevention in Children & Youth, 9(2), 192–218. 10.1080/15538340902824118 PMC2834206 20216916

[R35] Hosek S, & Pettifor A. (2019). HIV Prevention Interventions for Adolescents. Current HIV/AIDS Reports, 16(1), 120–128. 10.1007/s11904-019-00431-y 30707399 PMC6703904

[R36] Hussen SA, Chakraborty R, Camacho-Gonzalez A, Njiemoun B, Grossniklaus E, Goodstein E, Stephenson R, & Del Rio C. (2019). Beyond “purposeful and planned”: Varied trajectories of healthcare transition from pediatric to adult-oriented care among youth living with HIV. AIDS Care, 31(1), 45–47. 10.1080/09540121.2018.1488029 29897258 PMC9976190

[R37] Institute on Digital Health and Innovation. (2025). Youth engagement guide (Version 2.0). Florida State University: https://idhi.fsu.edu/sites/g/files/upcbnu4171/files/Youth%20Engagement%20Guide%20v2.0%20May%202025.pdf

[R38] Jaén J, Frankel A, French A, Davison R, Munoz-Laboy M, & Martinez O. (2024). Medical-Legal Partnerships: A promising approach for addressing health-harming legal needs among people with HIV. Frontiers in Sociology, 9, 1422783. 10.3389/fsoc.2024.1422783 39045387 PMC11264305

[R39] Johnson C, Chidester A, Chandramohan D, Lin H, Ho NM, Taranova A, Nijhawan AE, Kools S, Ingersoll K, Dillingham R, & Taylor BS. (2024). A call for youth voice to support engagement in care for 18- to 29-year olds living with HIV in the US South. AIDS Patient Care and STDs, 38(5), 238–248. 10.1089/apc.2024.0006 38662471 PMC11301709

[R40] Kapogiannis BG, Nelson RM, Siberry GK, Lee S, & Hazra R. (2018). Advancing HIV biomedical prevention research for at-risk adolescents. Journal of Acquired Immune Deficiency Syndromes, 79(5), 535–542. 10.1097/QAI.0000000000001853 30204722 PMC6231953

[R41] Klein MD, Beck AF, Henize AW, Parrish DS, Fink EE, & Kahn RS. (2013). Doctors and lawyers collaborating to help children—Outcomes from a successful partnership between professions. Journal of Health Care for the Poor and Underserved, 24(3), 1063–1073. 10.1353/hpu.2013.0147 23974381

[R42] Koay WLA, Aware Y, Andine T, Cruz Figueroa GM, Selekman RE, Bryant Y, & Rakhmanina NY. (2024). Patient perspectives on telehealth for HIV and mental health care at a pediatric and adolescent HIV clinic in Washington, DC. AIDS and Behavior, 28(3), 993–1001. 10.1007/s10461-023-04209-7 37843684

[R43] Krebs D, Goldhammer H, Dorfman M, Moore MP, Chavis NS, Psihopaidas D, Downes A, Bourdeau B, Saberi P, Grasso C, Mayer KH, & Keuroghlian AS. (2025). Telehealth interventions to improve HIV care continuum outcomes: A narrative review. AIDS Patient Care and STDs, 39(4), 129–140. 10.1089/apc.2024.0237 39929177

[R44] Lall P, Lim SH, Khairuddin N, & Kamarulzaman A. (2015). Review: An urgent need for research on factors impacting adherence to and retention in care among HIV-positive youth and adolescents from key populations. Journal of the International AIDS Society, 18(2 Suppl 1), 19393. 10.7448/IAS.18.2.19393 25724503 PMC4344535

[R45] LaLota M, Kwan BW, Waters M, Hernandez LE, & Liberti TM. (2005). The Miami, Florida, Young Men’s Survey: HIV prevalence and risk behaviors among urban young men who have sex with men who have ever runaway. Journal of Urban Health: Bulletin of the New York Academy of Medicine, 82(2), 327–338. 10.1093/jurban/jti056 15917503 PMC3456565

[R46] Landovitz RJ, Donnell D, Clement ME, Hanscom B, Cottle L, Coelho L, Cabello R, Chariyalertsak S, Dunne EF, Frank I, Gallardo-Cartagena JA, Gaur AH, Gonzales P, Tran HV, Hinojosa JC, Kallas EG, Kelley CF, Losso MH, Madruga JV, … Grinsztejn B. (2021). Cabotegravir for HIV prevention in cisgender men and transgender women. The New England Journal of Medicine, 385(7), 595–608. 10.1056/NEJMoa2101016 34379922 PMC8448593

[R47] Lee EOJ, Tang T-S, Fuentes-Bernal J, MacEntee K, Wachira J, Apondi E, Abramovich A, Oudshoorn A, Ayuku D, Kiptui R, Van Berkum A, MacDonald S-A, Saarela O, & Braitstein P. (2025). Optimal characteristics of peer navigators: Adapting peer-based intervention with street-involved youth in Canada and Kenya with the aim of increasing HIV prevention, testing and treatment. Health Research Policy and Systems, 23(1), 45. 10.1186/s12961-025-01309-9 40197481 PMC11974212

[R48] Lightfoot M. (2012). HIV prevention for adolescents: Where do we go from here? The American Psychologist, 67(8), 661–671. 10.1037/a0029831 23163452

[R49] Lightfoot M, Jackson-Morgan J, Pollack L, & Bennett A. (2022). Acceptability and feasibility of peer-to-peer text messaging among adolescents to increase clinic visits and sexually transmitted infection testing: Interrupted times-series analysis. JMIR Formative Research, 6(6), e32416. 10.2196/32416 35686737 PMC9227642

[R50] Liu Y, Chen A, Cho H, Siddiqi KA, Cook RL, & Prosperi M. (2025). Development of an electronic health record-based human immunodeficiency virus (HIV) risk prediction model for women, incorporating social determinants of health. BMC Public Health, 25(1), 2257. 10.1186/s12889-025-23460-2 40604593 PMC12219962

[R51] Lorenzetti L, Dinh N, van der Straten A, Fonner V, Ridgeway K, Rodolph M, Schaefer R, Schmidt H-MA, & Baggaley R. (2023). Systematic review of the values and preferences regarding the use of injectable pre-exposure prophylaxis to prevent HIV acquisition. Journal of the International AIDS Society, 26(Suppl 2), e26107. 10.1002/jia2.26107 37439057 PMC10805120

[R52] Lowenthal ED, Chapman J, Ohrenschall R, Calabrese K, Baltrusaitis K, Heckman B, Yin DE, Agwu AL, Harrington C, Van Solingen-Ristea RM, McCoig CC, Adeyeye A, Kneebone J, Chounta V, Smith-Anderson C, Camacho-Gonzalez A, D’Angelo J, Bearden A, Crauwels H, … Gaur AH. (2024). Acceptability and tolerability of long-acting injectable cabotegravir or rilpivirine in the first cohort of virologically suppressed adolescents living with HIV (IMPAACT 2017/MOCHA): A secondary analysis of a phase 1/2, multicentre, open-label, non-comparative dose-finding study. The Lancet. HIV, 11(4), e222–e232. 10.1016/S2352-3018(23)00301-6 38538161 PMC11061207

[R53] Lunkuse JF, Lwanga C, Wamono F, Muturi-Kioi V, Price M, & Mayanja Y. (2025). Willingness to use long-acting injectable pre-exposure prophylaxis among adolescent girls and young women in Kampala, Uganda. AIDS and Behavior, 29(5), 1458–1469. 10.1007/s10461-025-04616-y 39883369 PMC12031944

[R54] MacEntee K, Lee EOJ, Oudshoorn A, Abramovich A, Kiptui R, Ayuku D, Van Berkum A, Saarela O, Tang T-S, Apondi E, Wachira J, MacDonald S-A, & Braitstein P. (2022). Using scenario videos with Theatre Testing method to adapt a peer navigation model to improve street-connected youth’s access to HIV care in Kenya and Canada. Frontiers in Public Health, 10, 975117. 10.3389/fpubh.2022.975117 36408034 PMC9669244

[R55] Magno L, Leite BO, Grangeiro A, Dezanet L, Soares F, & Dourado I. (2025). Awareness and intention to use event-driven and long-acting injectable pre-exposure prophylaxis among adolescent and young men who have sex with men and transgender women in Brazil: A cross-sectional study. Journal of the International AIDS Society, 28(Suppl 2), e26479. 10.1002/jia2.26479 40600470 PMC12215819

[R56] Malow RM, Stein JA, McMahon RC, Dévieux JG, Rosenberg R, & Jean-Gilles M. (2009). Effects of a culturally adapted HIV prevention intervention in Haitian youth. The Journal of the Association of Nurses in AIDS Care: JANAC, 20(2), 110–121. 10.1016/j.jana.2008.12.003 19286123 PMC2694665

[R57] Marcus JL, Hurley LB, Krakower DS, Alexeeff S, Silverberg MJ, & Volk JE. (2019). Use of electronic health record data and machine learning to identify candidates for HIV pre-exposure prophylaxis: A modelling study. The Lancet. HIV, 6(10), e688–e695. 10.1016/S2352-3018(19)30137-7 31285183 PMC7152802

[R58] Martinez O, Munoz-Laboy M, & Davison R. (2022). Medical-legal partnerships: An integrated approach to advance health equity and improve health outcomes for people living with HIV. Frontiers in Reproductive Health, 4, 871101. 10.3389/frph.2022.871101 36303611 PMC9580720

[R59] Martinez O, Rodriguez S, Maude A, Brabson T, Fernandez I, Yang C, Davison R, French A, Nkwihoreze H, Momplaisir FM, Pozzi L, Alvarez D, McCallion P, Malave-Rivera S, Kingori C, Wu H, Liu F, Montanez A, Cruz-Gerena A, Morton S, & Munoz-Laboy M. (2026). Integrating legal aid into HIV care: Evaluating the impact of a medical-legal partnership on viral suppression outcomes. AIDS and Behavior, 30(2), 578–591. 10.1007/s10461-025-04901-w 41073583 PMC12579377

[R60] Martinez O, Rodriguez S, Maude A, Brabson T, Momplaisir FM, Nkwihoreze H, Davison R, French A, Pozzi L, Alvarez D, Malave-Rivera S, Cruz-Gerena A, Morton S, Rodriguez-Diaz C, & Muñoz-Laboy M. (2025). Justice as care: Embedding legal services into HIV care to address health-harming legal needs. Critical Public Health, 35(1), 1–14. 10.1080/09581596.2025.2575803 PMC12733083 41450764

[R61] Mayur SJ. (2026). Trust, fairness and safety in ethical artificial intelligence: A stakeholder theory-based conceptual framework. Advances in Consumer Research, 3(2), 619–622.

[R62] McCollister KE, Freitas DM, Prado G, & Pantin H. (2014). Opportunity costs and financial incentives for Hispanic youth participating in a family-based HIV and substance use preventive intervention. The Journal of Primary Prevention, 35(1), 13–20. 10.1007/s10935-013-0330-3 24162106 PMC3905081

[R63] McCree DH, Chesson H, Bradley ELP, Williams A, Gant Z, & Geter A. (2020). Exploring changes in racial/ethnic disparities of HIV diagnosis rates under the “ending the HIV epidemic: A plan for America” initiative. Public Health Reports^®^, 135(5), 685–690. 10.1177/0033354920943526 32762633 PMC7485057

[R64] Morris M, Handcock MS, Miller WC, Ford CA, Schmitz JL, Hobbs MM, Cohen MS, Harris KM, & Udry JR. (2006). Prevalence of HIV infection among young adults in the United States: Results from the add health study. American Journal of Public Health, 96(6), 1091–1097. 10.2105/AJPH.2004.054759 16670236 PMC1470623

[R65] Mulawa MI, LeGrand S, & Hightow-Weidman LB. (2018). eHealth to enhance treatment adherence among youth living with HIV. Current HIV/AIDS Reports, 15(4), 336–349. 10.1007/s11904-018-0407-y 29959649 PMC6086132

[R66] Murray A, Gaul Z, Sutton MY, & Nanin J. (2018). “We hide…”: Perceptions of HIV risk among black and Latino MSM in New York City. American Journal of Men’s Health, 12(2), 180–188. 10.1177/1557988317742231 PMC5818124 29161954

[R67] Nachega JB, Scarsi KK, Gandhi M, Scott RK, Mofenson LM, Archary M, Nachman S, Decloedt E, Geng EH, Wilson L, Rawat A, & Mellors JW. (2023). Long-acting antiretrovirals and HIV treatment adherence. The Lancet. HIV, 10(5), e332–e342. 10.1016/S2352-3018(23)00051-6 37062293 PMC10734401

[R68] Najjuuko C, Brathwaite R, Mutumba M, Childress S, Nannono S, Namatovu P, Lu C, & Ssewamala FM. (2025). Identifying predictors of problematic substance use among youth living with HIV in Uganda: A machine learning approach. AIDS and Behavior, 29(12), 4051–4062. 10.1007/s10461-025-04840-6 40965730 PMC13254183

[R69] Nwaozuru U, Miller L, Gunn LH, Marin-Cespedes S, Hanff M, Robinson P, Dulin M, Muralidhar M, Jha P, Mirikwe GC, Conserve DF, Gulden C, Davis BA, Foley K, Tucker J, & Zarwell M. (2025). Co-creating strategies to promote uptake of HIV self-testing among young adults in Mecklenburg county, North Carolina: A protocol for a pilot implementation study. Frontiers in Health Services, 5, 1536236. 10.3389/frhs.2025.1536236 40224904 PMC11985853

[R70] Ogunlana O, Kalulu P, Nwaozuru U, Olusanya OA, Olaoluwa OT, Ojo T, Gbaja-Biamila T, Arinze C, Akeem L, Fidelak L, Obi-Jeff C, Ezechi OC, Tucker JD, & Iwelunmor J. (2025). Digital strategies to promote crowdsourcing open calls for co-creating HIV interventions: A youth community-based participatory approach. Frontiers in Digital Health, 7, 1608366. 10.3389/fdgth.2025.1608366 40735342 PMC12303949

[R71] Okafor CN, Eaton L, & Watson R. (2024). Willingness to use long-acting injectable PrEP among PrEP Naïve Black and Hispanic sexual gender minority persons. AIDS and Behavior, 28(6), 2166–2174. 10.1007/s10461-024-04314-1 38526639

[R72] Oliwa T, Furner B, Schmitt J, Schneider J, & Ridgway JP. (2021). Development of a predictive model for retention in HIV care using natural language processing of clinical notes. Journal of the American Medical Informatics Association: JAMIA, 28(1), 104–112. 10.1093/jamia/ocaa220 33150369 PMC7810456

[R73] Pant Pai N, Kadam R, Jani I, Alemnji G, Malyuta R, & Peter T. (2025). The future of HIV diagnostics: An exemplar in infectious diseases. The Lancet. HIV, 12(7), e522–e531. 10.1016/S2352-3018(25)00078-5 40318692

[R74] Paone DL, Schuller JW, Smith ML, Gitlin LN, & Szanton SL. (2025). A 5-year examination of CAPABLE implementation using RE-AIM and CFIR frameworks. Frontiers in Public Health, 13, 1569320. 10.3389/fpubh.2025.1569320 40642250 PMC12243872

[R75] Perger T, Davtyan M, Foster C, Evangeli M, Berman C, Kacanek D, Puga AM, Sekidde S, & Bhopal S. (2025). Impact of HIV-related stigma on antiretroviral therapy adherence, engagement and retention in HIV care, and transition to adult HIV care in pediatric and young adult populations living with HIV: A literature review. AIDS and Behavior, 29(2), 497–516. 10.1007/s10461-024-04534-5 39453523 PMC11814060

[R76] Podschun GD. (1993). Teen peer outreach-street work project: HIV prevention education for runaway and homeless youth. Public Health Reports, 108(2), 150–155. 8464971 PMC1403354

[R77] Prado G, Huang S, Maldonado-Molina M, Bandiera F, Schwartz SJ, de la Vega P, Brown CH, & Pantin H. (2010). An empirical test of ecodevelopmental theory in predicting HIV risk behaviors among Hispanic youth. Health Education & Behavior, 37(1), 97–114. 10.1177/1090198109349218 20130302 PMC3715967

[R78] Proctor E, Silmere H, Raghavan R, Hovmand P, Aarons G, Bunger A, Griffey R, & Hensley M. (2011). Outcomes for implementation research: Conceptual distinctions, measurement challenges, and research agenda. Administration and Policy in Mental Health, 38(2), 65–76. 10.1007/s10488-010-0319-7 20957426 PMC3068522

[R79] Rajgopal PR, & Yadav S. (2025). The role of data governance in enabling secure AI adoption. International Journal of Sustainability & Innovation in Engineering (IJSIE), 3, 1–25.

[R80] Reid RJ, Lardier DT Jr., Garcia-Reid P, & Yu D. (2017). HIV testing among racial and ethnic minority adolescents living in an urban community. Journal of HIV/AIDS & Social Services, 16(3), 228–249. 10.1080/15381501.2016.1244653

[R81] Rice E, Tulbert E, Cederbaum J, Barman Adhikari A, & Milburn NG. (2012). Mobilizing homeless youth for HIV prevention: A social network analysis of the acceptability of a face-to-face and online social networking intervention. Health Education Research, 27(2), 226–236. 10.1093/her/cyr113 22247453 PMC3303208

[R82] Ridgway JP, Mason JA, Friedman EE, Oliwa T, Flores J, Simon J, Ekong A, Yang T-Y, & Schneider JA. (2025). Comparison of machine learning models to predict loss to follow-up among people with Human Immunodeficiency Virus (HIV). JAMIA Open, 8(4), ooaf077. 10.1093/jamiaopen/ooaf077 40709239 PMC12289141

[R83] Ritchwood TD, Malo V, Jones C, Metzger IW, Atujuna M, Marcus R, Conserve DF, Handler L, & Bekker L-G. (2020). Healthcare retention and clinical outcomes among adolescents living with HIV after transition from pediatric to adult care: A systematic review. BMC Public Health, 20(1), 1195. 10.1186/s12889-020-09312-1 32746881 PMC7398377

[R84] Ross J, & Stover J. (2013). Use of modern contraception increases when more methods become available: Analysis of evidence from 1982–2009. Global Health, Science and Practice, 1(2), 203–212. 10.9745/GHSP-D-13-00010 25276533 PMC4168565

[R85] Ryan AM, Kutob RM, Suther E, Hansen M, & Sandel M. (2012). Pilot study of impact of medical-legal partnership services on patients’ perceived stress and wellbeing. Journal of Health Care for the Poor and Underserved, 23(4), 1536–1546. 10.1353/hpu.2012.0179 23698668

[R86] Sabri B, Young N, Cardenas I, Emezue CN, & Patch M. (2026). Integrating implementation science in interpersonal violence research and practice: A systematic review of barriers and facilitators of implementation. Trauma, Violence & Abuse, 27(1), 41–65. 10.1177/15248380241305567 PMC12202711 39727243

[R87] Salinas JJ, & Valenzuela R. (2024). Using the reach effectiveness adoption implementation maintenance (RE-AIM) framework to evaluate a tailored education program to reduce obesity-related cancers in El Paso, Texas. International Journal of Environmental Research and Public Health, 21(8), 1051. 10.3390/ijerph21081051 39200661 PMC11354848

[R88] Schnall R, Kaw S, Pazol J, Brin M, Kuhns LM, Radix A, Naar S, & Garofalo R. (2026). An evidence-based approach to facilitating PrEP initiation among YMSM: The MyPEEPS plus mHealth and E-Peer navigation intervention. AIDS Education and Prevention, 38(2), 96–114. 10.1521/aeap.2026.38.2.96 42048232 PMC13310555

[R89] Serrano S, Wilton L, Sherpa D, Cleland CM, Zaldivar MF, Maria ZK, Rosmarin-DeStefano C, Munson MR, Padilla AS, & Gwadz M. (2025). Engaging diverse African American/Black and Latine youth and emerging adults living with HIV into research: Description of recruitment strategies and lessons learned. AIDS and Behavior, 29(1), 356–376. 10.1007/s10461-024-04524-7 39395069 PMC12977200

[R90] Setrini A. (2023). Using racial justice principles in medical-legal partnership design and implementation. The Journal of Law, Medicine & Ethics, 51(4), 757–763. 10.1017/jme.2023.159 PMC10937169 38477268

[R91] Spiegel JS, Salzman MS, Jones I, & Hacker L. (2023). Camden coalition medical-legal partnership: year one analysis of civil + criminal MLP model in addiction medicine setting. The Journal of Law, Medicine & Ethics, 51(4), 838–846. 10.1017/jme.2024.19 38477287

[R92] Stover J, Bollinger L, Izazola JA, Loures L, DeLay P, & Ghys PD. (2016). What is required to end the AIDS epidemic as a public health threat by 2030? The cost and impact of the fast-track approach. PloS One, 11(5), e0154893. 10.1371/journal.pone.0154893 27159260 PMC4861332

[R93] The National Center for Medical-Legal Partnership. (2018). Fact sheet: School-based health and medical-legal partnerships. https://www.medical-legalpartnership.org/mlp-resources/school-based-health/

[R94] Whitehead L, Robinson S, Arabiat D, Jenkins M, & Morelius E. (2024). The report of access and engagement with digital health interventions among children and young people: Systematic review. JMIR Pediatrics and Parenting, 7, e44199. 10.2196/44199 38231560 PMC10831666

[R95] Woodward EN, Matthieu MM, Uchendu US, Rogal S, & Kirchner JE. (2019). The health equity implementation framework: Proposal and preliminary study of hepatitis C virus treatment. Implementation Science, 14(1) N.PAG. 10.1186/s13012-019-0861-y PMC6417278 30866982

[R96] Wootton AR, Legnitto DA, Gruber VA, Dawson-Rose C, Neilands TB, Johnson MO, & Saberi P. (2019). Telehealth and texting intervention to improve HIV care engagement, mental health and substance use outcomes in youth living with HIV: A pilot feasibility and acceptability study protocol. BMJ Open, 9(7), e028522. 10.1136/bmjopen-2018-028522 PMC6661640 31315868

[R97] Zhang C, Kniffen W, Tang Y, & Liu Y. (2026). Leveraging artificial intelligence and nursing care to facilitate HIV prep utilization among black cisgender women: A qualitative study. Journal of HIV & Social Services, 24, 1–23. 10.1080/30681634.2026.2659659

